# Multiscale Feature Analysis of Salivary Gland Branching Morphogenesis

**DOI:** 10.1371/journal.pone.0032906

**Published:** 2012-03-05

**Authors:** Cemal Cagatay Bilgin, Shayoni Ray, Banu Baydil, William P. Daley, Melinda Larsen, Bülent Yener

**Affiliations:** 1 Rensselaer Polytechnic Institute, Computer Science Department, Troy, New York, United States of America; 2 University at Albany, State University of New York, Department of Biological Sciences, Albany, New York, United States of America; Centre for Genomic Regulation (CRG), Universitat Pompeu Fabra, Spain

## Abstract

Pattern formation in developing tissues involves dynamic spatio-temporal changes in cellular organization and subsequent evolution of functional adult structures. Branching morphogenesis is a developmental mechanism by which patterns are generated in many developing organs, which is controlled by underlying molecular pathways. Understanding the relationship between molecular signaling, cellular behavior and resulting morphological change requires quantification and categorization of the cellular behavior. In this study, tissue-level and cellular changes in developing salivary gland in response to disruption of ROCK-mediated signaling by are modeled by building cell-graphs to compute mathematical features capturing structural properties at multiple scales. These features were used to generate multiscale cell-graph signatures of untreated and ROCK signaling disrupted salivary gland organ explants. From confocal images of mouse submandibular salivary gland organ explants in which epithelial and mesenchymal nuclei were marked, a multiscale feature set capturing global structural properties, local structural properties, spectral, and morphological properties of the tissues was derived. Six feature selection algorithms and multiway modeling of the data was performed to identify distinct subsets of cell graph features that can uniquely classify and differentiate between different cell populations. Multiscale cell-graph analysis was most effective in classification of the tissue state. Cellular and tissue organization, as defined by a multiscale subset of cell-graph features, are both quantitatively distinct in epithelial and mesenchymal cell types both in the presence and absence of ROCK inhibitors. Whereas tensor analysis demonstrate that epithelial tissue was affected the most by inhibition of ROCK signaling, significant multiscale changes in mesenchymal tissue organization were identified with this analysis that were not identified in previous biological studies. We here show how to define and calculate a multiscale feature set as an effective computational approach to identify and quantify changes at multiple biological scales and to distinguish between different states in developing tissues.

## Introduction

Morphological and functional development of organs necessitates generation of multiple cell types and their coordinated spatio-temporal arrangement. Branching morphogenesis is a fundamental process controlling the growth and functional development of many mammalian exocrine glands such as the lung, kidney, pancreas, prostate glands, mammary glands and salivary glands [1]. During development of major exocrine organs, the process of branching morphogenesis was adopted to satisfy the requirement for efficient exchange of gases, nutrients, metabolites, and wastes with the environment. Branching morphogenesis enables packing of a large surface area of epithelium into a relatively small volume, thereby increasing the surface area in contact with the environment. Important questions regarding the signals controlling branching, what patterns are followed by the organs, and how these movements are regulated at cellular and tissue level are just beginning to be explored. Recent studies in another organ that undergoes branching morphogenesis, the developing lung, identified a set of three stereotypical geometric subroutine patterns that when reiteratively combined result in an adult lung [2]. The branching pattern in the developing salivary gland is different than in the lung since the gland undergoes a series of cleft formation events rather than the bifurcation events that occur during lung development [3]. Since the branching pattern in salivary gland is different and the morphological patterns are less apparent at the tissue level than in the lung, we investigated whether a computational approach could be used to identify, quantify, and specify the cellular and tissue level organization of developing salivary glands as a first step in understanding the processes controlling organogenesis.

In the past several years, mapping out interconnectedness within systems, or ‘Network analysis’, has revolutionized our understanding of complex events that function not only at various scales but with a multitude of players involved in multiple events. The structure and function of multiple types of networks ranging from internet-based social networks to biological networks can be modeled by graphs. These graph theoretical models have been used to extract information about the function of complex biological networks, from protein-protein interactions [4],[5], disease progression [6], metabolic networks [7],[8], genetic and transcriptional regulatory systems [9], and neuronal connectivity [10]. These studies have provided important insights into the construction and function and regulation of these networks on both global and local scales.

Network analysis is primed to decipher cellular interactions, since cellular events comprise an intricate interplay between protein-protein interactions, genetic changes, metabolic pathways and chemical secretions. When extended at an organ level, the key challenge would be to link local and global structural properties of tissues to the overall morphology and function of a tissue. Only a systems level understanding of the various cellular processes at multiple biological levels will take into account the multi-dimensional complexity of these processes. If the principles governing biological organization in a morphological, spectral, local and global scale can be deduced, the correlation between structural and molecular signaling within the tissue can be understood and be applied to inform and accelerate studies of organ development and tissue regeneration.

In previous work [11]–[16], we developed a graph theoretical method called cell-graphs to model cellular networks to classify features in human pathological specimens. Cell-graphs capture the characteristic structural properties that distinguish healthy, damaged, and cancerous states of brain, breast, and bone tissues [11]–[13]. We further extended this method to model mesenchymal stem cells in three dimensional space [14], to ECM interactions during cell-mediated compaction and collagen remodeling in 3D [15]. We also showed preliminary results of the applicability of cell-graph technique for capturing the distinctive epithelial and mesenchymal features in an embryonic branching organ – the salivary gland [16]. Application of graph theory to cellular networks provides a rich set of computational features that represent the structural characteristics of the underlying tissue samples. Cell-graphs are generalizations of Delaunay Triangulation used to model spatial distribution of cells in a tissue by encoding a pair-wise spatial relationship between them [17],[18]. In a cell-graph, vertices (or nodes) represent cell nuclei and pairs of vertices are connected by an edge (or a link), determined according to a theoretical biological relationship between them which may represent either a chemical or a physical association. These studies demonstrated that two classes of cell graph features, global-structural and spectral, can capture unique feature descriptions for distinct tissue states.

We previously identified Rho associated coiled-coil kinase 1 (ROCK1), a serine-threonine kinase that is activated downstream of Rho GTPase, to be a critical regulator of branching morphogenesis in mouse salivary gland and demonstrated that ROCK1 has a critical function in regulating morphological change. ROCK1 regulates progression of clefts, or indentations, in the smooth surface of the primary epithelial bud during branching morphogenesis. We demonstrated that ROCK1 alters organ shape by altering actin-myosin mediated contractility, which is required for assembly of fibronectin in the basement membrane during cleft progression [19] and regulation of focal adhesion formation in the outer epithelial cell layer [20]. Additionally, ROCK stimulates changes in the cellular organization [21].

In the current study, we developed a cell-graph-based multiscale feature analysis capturing changes in cellular behavior and resulting organ shape upon treatment with ROCK1 inhibitor; thus providing insight into the cellular dynamics of submandibular gland (SMG) morphogenesis and the function of ROCK1-mediated signaling in this process. We investigated the utility of cell-graphs to understand the relationship between cellular-, tissue-, and organ-level changes in response to molecular signaling. To accomplish his, we developed new cell-graph feature sets capturing the local characteristics of nodes and the morphological properties of the tissues. The addition of these two different scales made it possible to interrogate cellular, tissue, and organ shape changes using a multi-scale analysis of salivary gland tissues in response to disruption of ROCK signaling.To perform such a multiscale study, we provide correlation analysis within and between the scales, with and without ROCK1 treatment. We also performed a 3-way tensor analysis to find underlying cellular patterns. As in our previous work we also test our modeling using classification and feature selection methods to identify the cell graph features most representative of cellular-, tissue-, and organ-level changes.

## Results

### Immunostaining and Image Acquisition

**Figure 1:**
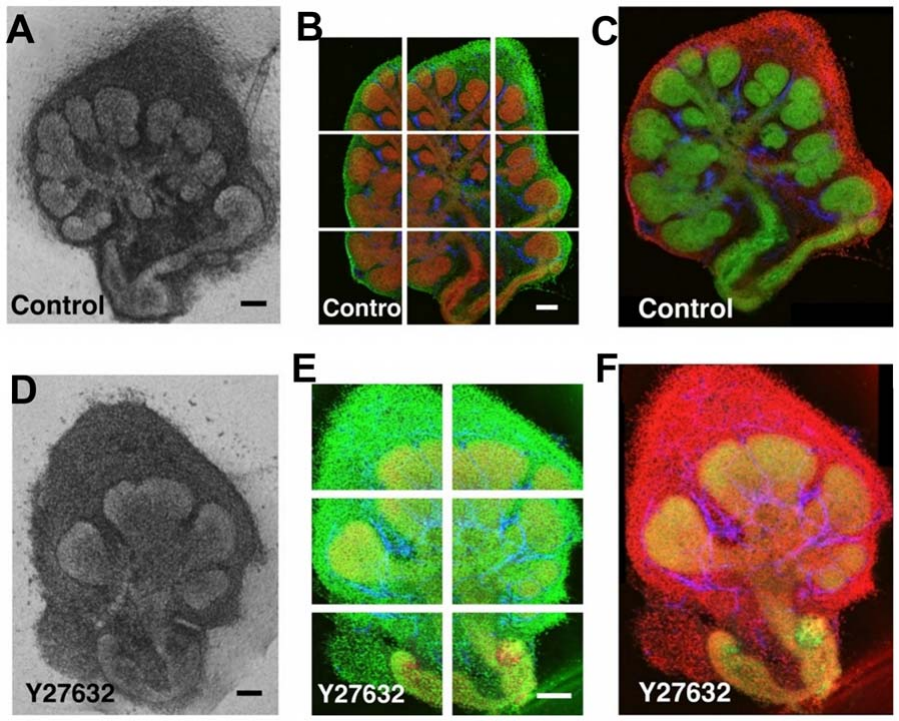
Acquisition and image processing of confocal images. Organotypic culture of E13 SMGs (a) control or (b) treated with ROCK inhibitor (140 µM Y27632), showing reduced branching with ROCK inhibitor treatment. Explants were immunostained with anti-E-cadherin antibody as an epithelial marker (red) and SYBR green as a total nuclei marker (green). Multiple overlapping confocal images through the mid-section of (c) control- and (d) ROCK inhibitor-treated explants were captured to cover the whole explant. Images were stitched using the inverse Fourier transform of the phase correlation matrix and blended to provide composite images of (e) control (f) and ROCK inhibitor treated explants. Scale bars: 200 µm (a, b), 100 µm (c), (d), and (e), and (f). In our study, the sublingual tissues were discarded and only the submandibilar gland was used, (Figure S2).

We probed for quantitative changes in mouse embryonic submandibular gland (SMG) organ explants that were treated with the ROCK1/2 inhibitor, Y27632, using cell-graph methods. To do this, embryonic E13 SMGs were cultured ex-vivo for 24 hours in the absence or presence of ROCK inhibitor, as shown in Figure 1A, D. They were treated with Sybr Green total nuclei marker (green) to detect total nuclei and immunostained with an anti-E-cadherin antibody as an epithelial marker (red) to identify epithelial cells. Cells not expressing the cell-cell adhesion protein E-cadherin were classified as mesenchymal cells. Multiple overlapping confocal images were captured from the center of each explant at 20× magnification (Figure 1B, E). Each dataset consists of 20 samples of vehicle control- and ROCK inhibitor-treated organ explants each.

### Image Registration and Nuclei Segmentation

**Figure 2:**
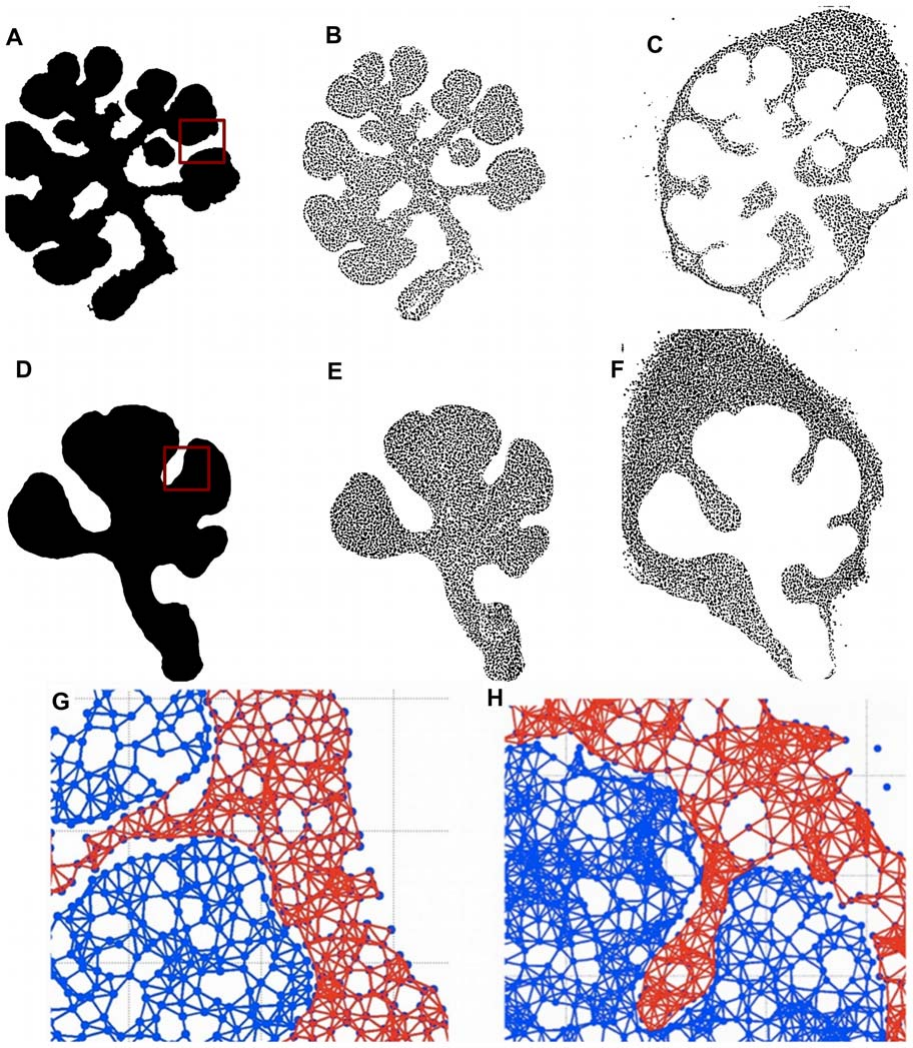
Generation of Cell Graphs. Stitched images were segmented using the active contour method to define epithelial (white) vs mesenchymal tissue (black) in control (a) and ROCK inhibitor-treated explants (d). These masks were used to identify the epithelial nuclei (b, e) and mesenchymal nuclei (c, f). Using each nucleus as a vertex, cell-graphs were constructed for control and ROCK inhibitor-treated tissues, respectively (g, h), where zoomed regions of cell graphs corresponding to regions of the original images (shown as red boxes in a and d) are shown in detail. Epithelial tissue is respresented by the blue graph and the mesenchymal tissue is represented by the red graph. We discarded the sublingual tissues and only used the submandibilar gland, (Figure S2).

Segmentation was performed on composite images representing entire organ explants. Overlapping confocal images were computationally stitched together to generate composite images (Figure 1C, F). These stitched images were segmented to identify the epithelial and mesenchymal regions using active contours without edges technique (Figure 2A, D). Nuclear segmentation was performed using the Otsu thresholding algorithm followed by the Watershed technique [22]. The results of the nuclei segmentation for control- and ROCK1 inhibitor-treated epithelial and mesenchymal tissues are shown in Figure 2B, C, E and F, respectively.

### Cell-Graph Construction and Feature Extraction

Cell-graph construction captures the pair-wise distance relationship between the cells to provide a structural modeling of the tissue. Formally, a graph, *G*, is represented by *G = (V,E)*, where *V* is the vertex set and *E* is the edge set of the graph. In the cell-graph representation of each tissue [11]–[16] each cell constitutes a vertex in the graph, and an edge is set between two cells having coordinates 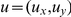 and 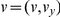 if the Euclidean distance between them, 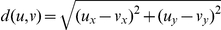, is considered small enough to facilitate communication between these two cells. When the dataset is large, cross-validation techniques can be used to identify the optimal threshold that might signify cell-cell communication. In cases when the dataset is limited in size, heuristics such as five times the average radius of a nucleus can be used. A typical heuristic threshold value used in our modeling is 20 microns. The optimal threshold can be verified by a visual comparison of the cell graph with the confocal image. In Figure 2G, H, cell-graph modeling of both the epithelial and mesenchymal tissue are depicted for control and ROCK inhibitor-treated tissue samples, respectively.

### Generation of New Features and Feature Extraction

From cell-graphs, quantitative metrics can be extracted to predict relationships between the cells. A rich set of features was computed from these cell-graphs, capturing multiple levels of relationships between the cells and structural characteristics of the tissue. Features capturing global structural characteristics between the cells were calculated (Table 1). Spectral analysis of cell-graphs was included to represent the spectral scale of the cell-graphs (Table 2). Local structural features (Table 3) capturing the local interactions between the cells were also included. Shape-based features of the epithelial tissues were extracted to capture the morphological properties of tissues (Table 4). That is, the multi-scale features span four different categories forming the basis for a multiscale feature analysis of the organ properties.

**Table 1:** Global structural features.

Feature Index	Feature Name	Feature Explanation
Connectedness and Cliquishness Measures:		
1	Average Degree	Average value of number of neighbors a node has.
2	Clustering Coefficient (C) of a Node	The ratio of the links a node's neighbors have in between to the total number that can possibly exist.
3	Clustering Coefficient (D) of a Node	The ratio of the links a node's neighbors have in between to the total number that can possibly exist.
4	Clustering Coefficient (E) of a Node	The ratio of the links a node's neighbors have in between to the total number that can possibly exist.
12	Giant Connected Component Ratio	Ratio of the size of the largest set of the vertices that are reachable from each other to the number of vertices.
13	Number of Connected Components	Total number of components that are reachable from each other.
14	Percentage of Isolated Points	The ratio of number of vertices with degree equal to zero
15	Percentage of End Points	The ratio of number of vertices with degree equal to one to the total number of vertices.
Distance Based (Shortest-path related) Features:		
-	Eccentricity of a Node	Maximum value of the shortest path from a given node to any other node.
5	Average Eccentricity	Average value of the eccentricity values for all the vertices.
6	Diameter	Maximum eccentricity.
7	Radius	Minimum eccentricity.
8	90 percent reachable Average Eccentricity of a Node	Maximum value of the shortest path from a given node to any other node.
9	90 percent Diameter	Maximum eccentricity.
10	90 percent Radius	Minimum eccentricity.
11	Closeness of a Node	Average value of the shortest path from a given node to any other node.
16	Number of Central Points	Number of vertices that have eccentricity equal to radius.
17	Percent of Central Points	Percentage of vertices that have eccentricity equal to radius.
18	Number of Vertices	Number of cells in the tissue.
19	Number of Edges	Number of hypothesized communications.

**Table 2:** Spectral features.

Feature Index	Feature Name	Feature Explanation
20	Largest eigenvalue adjacency	Largest valued eigenvalue
21	Second Largest eigenvalue adjacency	Second largest valued eigenvalue
22	Trace of adjacency	Sum of the eigenvalues of the adjancency matrix.
23	Energy of adjacency	Squared sum of the eigenvalues of the adjancency matrix.
24	Number of zeros normalized Laplacian	Number of eigenvalues that are 0.
25	Lower Slope	The slope of the line for the eigenvalues that are between 0 and 1 when sorted and plotted.
26	Number of ones normalized Laplacian	Number of eigenvalues that are 1.
27	Upper Slope	The slope of the line for the eigenvalues that are between 1 and 2 when sorted and plotted.
28	Number of twos normalized Laplacian	Number of eigenvalues that are 2.
29	Trace of Laplacian	Sum of the eigenvalues
30	Energy of Laplacian	Squared sum of the eigenvalues

**Table 3:** Local structural features.

Feature Index	Feature Name, i = {1,2 3}	Feature Explanation
31–33	Degree of the i^th^ representative vertex	Average number of neighbors for the i^th^ representative node
34–36	Clustering coefficient C of the i^th^ representative vertex	The ratio of the links of the i^th^ representative node's neighbors have in common to the total number that can possibly exist
37–39	Clustering coefficient D of the i^th^ representative vertex	The ratio of the links of the i^th^ representative node's neighbors have in common to the total number that can possibly exist
40–42	Clustering coefficient E of the i^th^ representative vertex	The ratio of the links of the i^th^ representative node's neighbors have in common to the total number that can possibly exist
43–45	Eccentricity of the i^th^ representativeI vertex	Maximum value of the shortest path values from the i^th^ representative node
46–48	Effective eccentricity of the i^th^ representative	Maximum value of the 90% reachable shortest path values from the i^th^ representative node
49–51	Closeness of the i^th^ representative	Average value of the shortest path values from the i^th^ representative node
52–54	Betweenness of the i^th^ representative	The number of times that i^th^ representative node occurs on a shortest path
55–57	Mean *knn* distance of the i^th^ representative	The mean of the physical distances between the i^th^ representative node and the nodes that are k hop apart from it (*knn*: k nearest neighbourhood)
58–60	Standard deviation of the *knn* of i^th^ representative	The standard deviation of the physical distances between the i^th^ representative node and the nodes that are k hop apart from it
61–63	Skewness of the *knn* of i^th^ representative	The skewness of the physical distances between the i^th^ representative node and the nodes that are k hop apart from it
64–66	Kurtosis of the *knn* of the i^th^ representative	The kurtosis of the physical distances between the i^th^ representative node and the nodes that are k hop apart from it
67–69	Mean of the physical *knn* distance of the i^th^ representative	The mean of the physical distances between the i^th^ representative node and the nodes that are at k times the link threshold distance from it
70–72	Standard deviation of the physical *knn* distance of the i^th^ representative	The standard deviation of the physical distances between the i^th^ representative node and the nodes that are at k times the link threshold distance from it
73–75	Skewness of the physical *knn* distance of the i^th^ representative	The skewness of the physical distances between the i^th^ representative node and the nodes that are at k times the link threshold distance from it
76–78	Kurtosis of the physical *knn* distance of the i^th^ representative	The kurtosis of the physical distances between the i^th^ representative node and the nodes that are at k times the link threshold distance from it
79–81	Mean edge length of the i^th^ representative	Mean edge length of the i^th^ representative node to its neighbors
82–84	Standard deviation of the edge length of the i^th^ representative	Standard deviation of the edge length of the i^th^ representative node to its neighbors
85–87	Skewness of the edge length of the i^th^ representative	Skewness of the edge length of the i^th^ representative node to its neighbors
88–90	Kurtosis of the edge length of the i^th^ representative	Kurtosis of the edge length of the i^th^ representative node to its neighbors
91–93	Number of hybrid edges of i^th^h representative	For an epithelial cell, the number of mesencyhmal cells that it is connected to

**Table 4:** Morphological (shape based) features.

Feature Index	Feature Name	Feature Explanation
94	Elongation	The ratio of major axis length to minor axis length
95	Area	The number of pixels in the region
96	Orientation	The angle between the x-axis and the major axis of the region.
97	Eccentricity	The ratio of the distance between the foci of the ellipse and its major axis length.
98	Perimeter	The distance around the boundary of the epithelial region.
99	Circularity	Perimeter squared over 4*Area
100	Solidity	The ratio of the area to the convex hull area
101	Fractal Dimension	The limit of the ratio of ln(N) to ln(s) as s goes to zero where N is the number of boxes with side s that covers the shape

We developed a rich set of features from different scales to represent the branching morphogenesis from different perspectives. Our previous feature set was mainly confined to global structural features. Using this global structural feature set we modeled and classified brain [11], breast [12], bone [13] and salivary gland [16] tissues. However, in this study we developed new features to address the patterns in salivary gland development and capture the multiscale aspect of it. Specifically, we introduced local structural and morphological features to analyze the local behavior and shape characteristics of the tissues. We implemented the degree, clustering coefficient, eccentricity, effective eccentricity, closeness, betweenness, k-nearest neighborhood distance statistics, physical k-nearest neighborhood distance statistics, edge length statistics and number of hybrid edges for local structural modeling.

The distances between a node and the nodes that are k hop apart from it are calculated and mean, standard deviation, skewness and kurtosis of these distances are measured and called the k-nearest neighborhood (*knn*) distance statistics. Around the clefts this distance is expected to be small compared to other parts of the tissue. As the ROCK inhibitor-treated examples have a greater number of small, initiated clefts, this local region in these tissues will have greater *knn* (k-nearest neighborhood) values and, therefore, *knn* values might be a good candidate for classification between ROCK inhibitor-treated and untreated samples. A slightly different version of this feature is also included in the analysis, namely physical k-nearest neighborhood distance. In the calculation of this feature, instead of using nodes that are k-hop apart, nodes that are k times the link threshold distance apart from each other are used. Statistics of this feature such as the first, second, third and fourth moments (mean, std, skewness and kurtosis) were included. Another feature we developed specifically for this analysis is the number of hybrid edges. For an epithelial cell, the number of mesencyhmal cells that it is connected to is also calculated and used as the number of hybrid edges. Using these features, the local view of the tissue is modeled and tissues are classified according to their local interactions. A detailed description of how these features are calculated is given in the materials and methods section.

### Cell-Graph Calculations and Biological Validation

**Figure 3:**
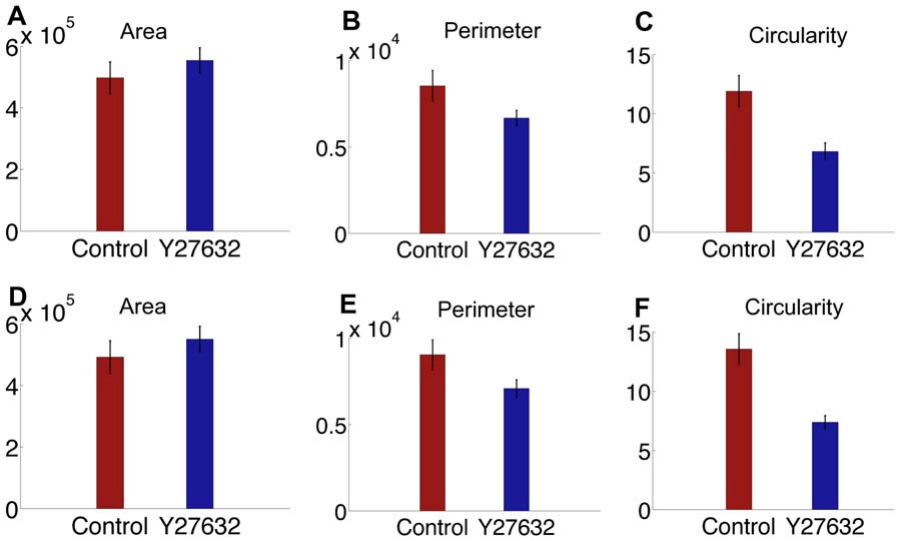
Direct validations of cell-graph features using standard image analysis methods. Plots of (a) area, (b) perimeter and (c) circularity from images using conventional image analysis methods and plots of cell-graph-derived raw data pertaining to (d) area, (e) perimeter and (f) circularity are shown. Control refers to untreated epithelium and Y27632 refers to the ROCK inhibitor treatment. The same trends for control vs ROCK inhibitor treatment were observed for the features obtained using image analysis and cell-graphs. The percent differences between the conventional image analysis and our image segmentation technique are found to be 1.16% and 0.73% for the area; 5.66% and 5.94% for the perimeter; 11.0532 and 16.1463 for the circularity of the control and ROCK inhibitor-treated samples, respectively.

With any computational method, it is necessary to validate computational results, whenever possible, with results obtained more directly from the sample. Therefore, after extracting the full set of cell graph features, we compared the values of a subset of these cell-graph features to the corresponding values obtained using conventional image analysis methods directly on the confocal images and validated the cell-graph measurements. We calculated the average area, perimeter, and circularity and the standard error of the organ explants using standard image processing methods directly from the confocal images for each treatment, as shown in Figure 3A–C and directly compared these results with the values for the same features derived from the morphological analysis. The same trends were observed for this subset of features in control vs ROCK inhibitor-treatment for the conventional and computational analysis, calculated only for the epithelial tissue.

**Figure 4:**
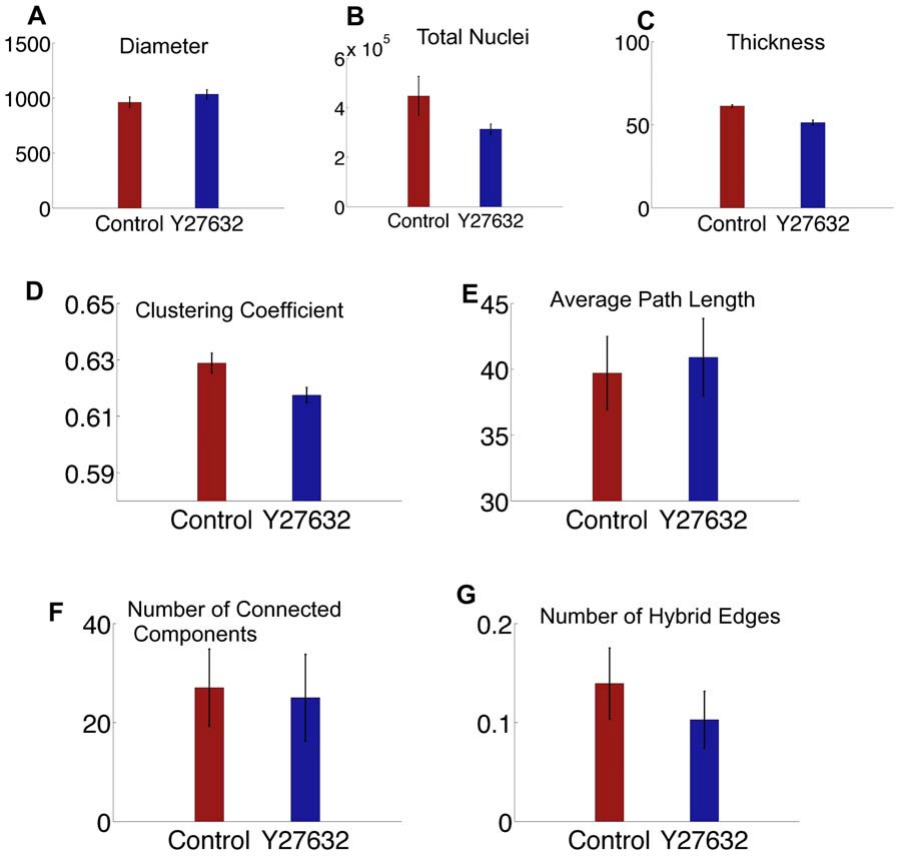
Indirect validations of cell-graph features using standard image analysis methods. Control refers to untreated epithelium and Y27632 treated epithelium. (a) Diameter of explants was measured using MetaMorph image analysis tools from single confocal images (b) Total nuclei were measured from single confocal images. (c) Thickness was measured from confocal Z-stacks of images. With Y27632 treatment, diameter of the explants increases and thickness and number of cells decreases thus reducing the overall compactness of the tissue structure. Cell-graph-derived features, such as clustering coefficient (d), average path length (e) and number of connected components (f) show that Y27632 treatment increases the distance between two cells, thereby lowering the number of linked cells and decreasing the overall compactness in the epithelial and mesenchymal regions.

We previously observed that treating SMGs with the ROCK inhibitor, Y-27632, an alternate ROCK inhibitor, H-1152, or ROCK1 siRNA caused a decrease in intracellular contractility and a subsequent decrease in cell proliferation [19]. Using conventional image analysis methods, we verified that the average diameter of the SMG increased and that the thickness decreased following inhibitor treatment (Figure 4A), as we previously reported [19], which are consistent with the overall decrease in cellular contractility. Additionally, we verified that the total number of cells also decreased with inhibitor treatment (Figure 4B). This led us to predict that the overall compactness of the explant decreases both at the tissue and at the cellular level with ROCK inhibitor-treatment, which should be measurable using specific cell-graph features. The values for cell-graph features indicated that with ROCK inhibitor treatment, the clustering coefficient in the control tissues was greater than in the ROCK inhibitor-treated tissues. The clustering coefficient, gives a measure of compactness of a tissue. That is, cells in the ROCK inhibitor-treated tissues were further apart from each other and thus, had fewer edges, or links, per unit area, measurable as a decreased clustering coefficient. The average path length, which measures the average shortest path between two cells (Figure 4E), increases with ROCK inhibition and number of connected components (Figure 4F), which is the number of cell-linked cell clusters, decreases. If the tissue is less compact, it should have a smaller number of linked cells, an increased inter-cellular distance (longer average path length) and, hence, a lower number of connected components. Cell-graph features were thus able to predict known ROCK inhibitor-induced global tissue changes.

We also observed local changes in tissue structure upon treatment when ROCK was inhibited,. In the presence of the ROCK inhibitor, Y27632, or in the presence of ROCK1 siRNA but not ROCK2 siRNA, the outer columnar cell layer that lines the periphery of the buds is disorganized [21] (Figure S1B) compared to the untreated control SMGs (Figure S1A). This difference in cellular organization was detectable as an decrease in the number of hybrid edges – which represents the link between epithelial and mesenchymal cells – in ROCK inhibitor-treated SMG compared to untreated control SMGs (Figure 4G). This data indicates that cell-graphs are capable of detecting local subtle changes in tissue organization.

### Feature Correlation Analysis

We perform feature correlation analysis to observe how the multi-scale relations for the epithelial and mesenchymal tissues differ with and without inhibitor treatment. For all tissue samples cell-graph features were clustered into four groups, based on the similarity of their (signed) correlations with the whole cell-graph feature set, using the k-means clustering algorithm. The optimal number of clusters was found to be k = 4, as there are four different scales. In Figure S3 A–D, re-grouped correlation maps of control epithelial, control mesenchymal, ROCK inhibitor-treated epithelial and ROCK inhibitor-treated mesenchymal tissues are provided, respectively. The subset (5–11) of the shortest path related features (5–11, 16–19) fall into the same correlation cluster in all the four tissue samples (Figure S3A–D); and while these correlation clusters contain the subset (22, 23, 29, 30) of the spectral feature set (20–30) in the three tissue samples, control epithelial, ROCK inhibitor-treated epithelial and control mesenchymal, (Figure S3A–C respectively), this is not the case in ROCK inhibitor-treated mesenchymal tissue (Figure S3D). That is, the multi-scale correlations between the spectral features and the shortest path features disappear in the ROCK inhibitor-treated mesenchymal tissues.

In epithelial tissues, the cluster that holds the shortest path-related features (5–11) and the subset (22, 23, 29, 30) of the spectral features (20–30) also holds the subset (95, 98, 99) of the local features (31–93), as observed in a comparison of Figure S3A and Figure S3B, This suggests that the global structural features, a subset of the spectral features and a subset of the local structural features are correlated with each other, and that this correlation is not affected by the ROCK inhibitor-treatment. Likewise, comparison of Figure S3A and Figure S3B shows that in epithelial tissue, the correlation cluster that holds most of the local structural features (31–93) is preserved in the ROCK inhibitor-treated epithelial tissues. That is, the correlation cluster of the subset (37, 43–47, 49, 50, 55–60, 64–66, 71, 79, 81, 88, 89, 90) of the local feature set is the same for both the ROCK inhibitor treated and untreated epithelial tissue samples. This suggests that the correlation structures of these features are independent of the treatment and that the inhibitor treatment does not affect the multi-scale relation of local features in epithelial tissues

### Feature correlation cluster changes

**Figure 5:**
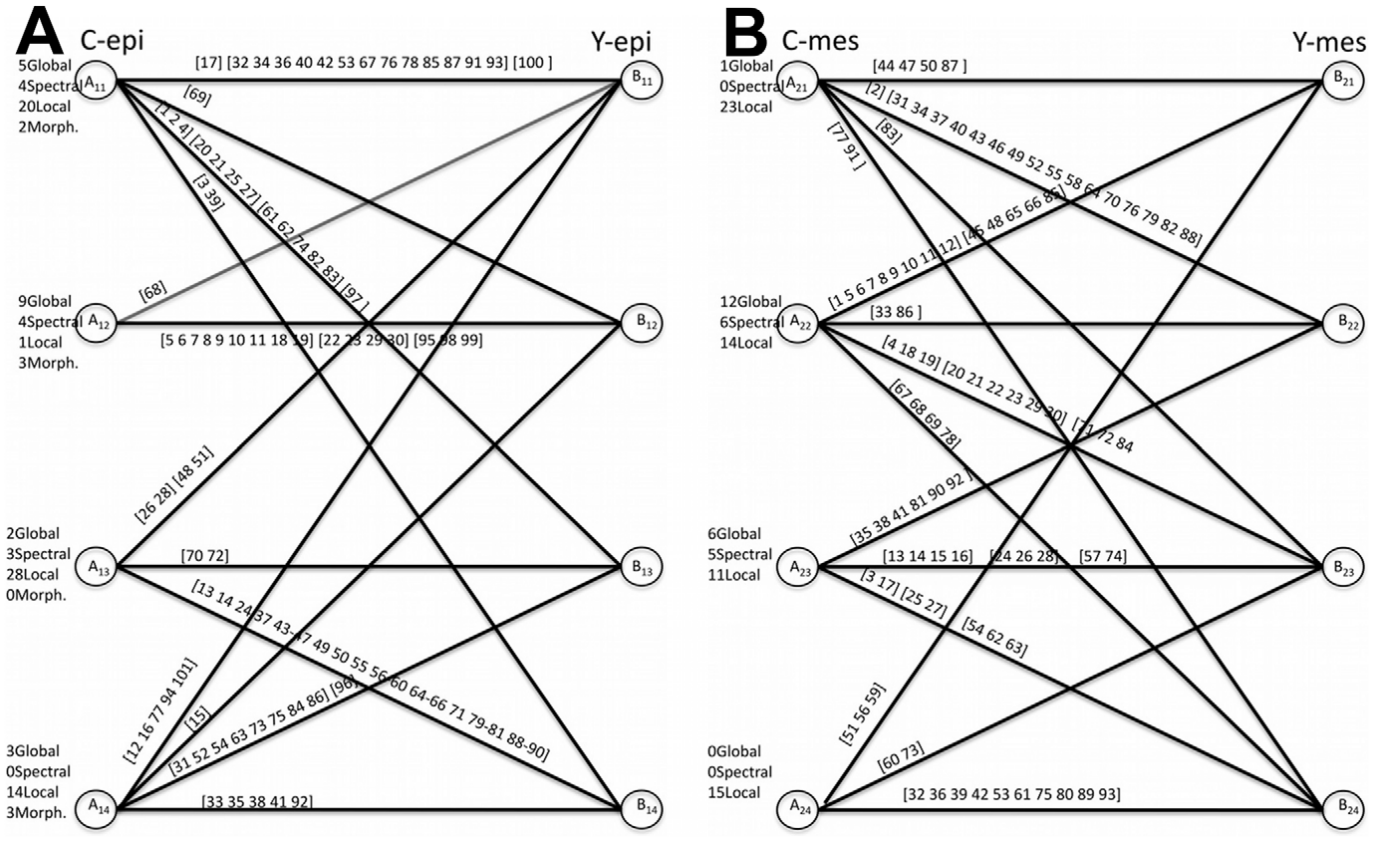
Bipartite graph analysis. The changes in the correlation clusters of the four tissue samples are studied through bi-partite graph analysis for the untreated vs. treated epithelial tissue comparison in (a) and for the untreated vs. treated mesenchymal tissue comparison in (b).

The changes in the correlation clusters, which were found by the k-means algorithm in the previous section, are studied in a systematic way through bi-partite graph analysis, as shown in Figure 5A and B. Here, a link between two clusters of the compared tissue samples means that there is at least one common cell-graph feature in the two linked correlation clusters and the indices of the common cell-graph features are written above the links connecting the correlation clusters. Indices of the features that are in the same feature category are grouped together in the same bracket. For each correlation cluster, the number of features it contains from each of the four feature categories, global structural, morphological shape-based, spectral, and local structural, are also shown next to the correlation cluster.

The bi-partite graph analysis indicates that ROCK inhibitor-treatment affects the correlation cluster structure of cell-graph features of the epithelial and mesenchymal tissues equally but differently: the number of links in Figure 5A and B differs by one, meaning they are affected almost equally by the treatment. However, the features that are affected, as depicted on the bipartite graph edges, within each correlation cluster differ. Through Figure 5A–B, feature subsets that are in the same correlation clusters in both control epithelial and ROCK inhibitor-treated epithelial tissue samples can be identified (from Figure 5A) and how this correlation cluster structure differs in the case of mesenchymal tissues (from Figure 5B) can be tracked. For instance, while the subset (5–11,18,19) of the global structural feature set (1–19) and the subset (22,23,29,30) of the spectral feature set (20–30) continue to be in the same correlation cluster after ROCK inhibitor-treatment in epithelial tissue (from Figure 5A); these two subsets of features, which also belong to one correlation cluster in control mesenchymal tissue, are distributed over two different links in Figure 5B), meaning they belong to two different correlation clusters in ROCK inhibitor-treated mesenchymal tissue. This suggests that the multi-scale relationship between the global-structural features and the spectral features is preserved in epithelial tissues with the treatment, whereas in the mesenchymal tissues this relationship disappears with the ROCK-I inhibitor treatment.

Another difference between the effect of the treatment on the correlation clusters is that the second correlation cluster in the control epithelial tissues is preserved with little changes in the ROCK inhibitor treated epithelial tissues, e.g. in the control epithelial tissue feature indexed with 68 belongs to the second correlation cluster whereas in the treated case this feature is dropped from the cluster and 15 and 69 are added to the same cluster. However, in the comparison of mesenchymal tissue, all the correlation clusters change after the inhibitor treatment. That is, the changes in mesenchymal tissue correlation clusters are more diverse and the multi-scale relationships in mesencymal tissues change more compared to the epithelial tissues with the treatment.

### Significant feature correlations

To analyze only the significant relationships of the four feature categories to each other, we applied statistical analysis to the pairwise feature correlation results to identify correlations having 95% significance. The four correlation maps provided in Figure S4A–D show only correlations having 95% or greater significance. The entries in these correlation maps are ordered in parallel with the ordering of the indices in Table 1, 2, 3, 4 to facilitate identification of significant correlations between the four feature categories in each tissue sample. The changes that occur in correlations between the feature categories under ROCK inhibitor-treatment of epithelial and mesenchymal tissues are also analyzed. In Figure S4A–D, the lower left corners correspond to tissue connectedness and cliquishness features (1–4, 12–15) and to shortest path-related features (5–11, 16–19) and the right top corners correspond to local structural features (31–93) and shape-based morphological features (94–101). The most informative values in these correlation maps are located in the off-diagonal entries. The correlations are provided as absolute values, meaning that a value of 1 indicates either a perfect positive correlation or a perfect negative correlation. In this and the remaining sections, indices of the referred to features will be indicated in parenthesis.

### Feature correlation analysis between epithelial and mesenchymal tissues

Comparison of the control epithelial and mesenchymal tissue feature correlation maps (Figure S4A and Figure S4C respectively) shows indeed that while the spectral-based feature set (20–30) lacks significant correlation with the other feature categories in the mesenchymal tissue, in the epithelial tissue, it is significantly correlated with other feature categories, such as the subset (5–11) of the shortest path-related features (5–11, 16–19) and the local structural feature set (31–93).

In the case of the ROCK inhibitor-treated epithelial tissue versus inhibitor-treated mesenchymal tissue correlation comparison, the number of significant correlations within the spectral feature set is higher for the mesenchymal tissues. Moreover, the subset (5–11) of the shortest path-related features (5–11, 16–19) is more correlated with the subset (40–50) of the local structural feature set in the case of inhibitor-treated mesenchymal tissue. While the spectral feature set lacks significant correlation with the local structural feature set (31–93) in the inhibitor-treated epithelial tissue, the correlations between these two sets of features increase in the inhibitor-treated mesenchymal tissue.

Feature correlation analysis in epithelial tissue: After treatment of the epithelial tissue with ROCK inhibitor, a reduced correlation between the local structural feature set (31–93) and spectral feature set (20–30) is observed (Figure S4B). Here, a reduced correlation between two sets refers to a decrease in the number of pairwise correlated features and/or a decrease in the absolute value of correlation coefficients across the referred sets. Similarly, the number of pairwise significant correlations within the local structural feature set (31–93) decrease with the ROCK inhibitor-treatment. Apart from these, an increase in the correlation of the global structural features (1–19) is observed. Likewise, shape-based features (94–101) correlate with each other more in the presence of the ROCK inhibitor-treatment. An increase in the correlation of the global structural features and local structural features is also observed under these conditions. After treatment of the epithelial tissue with ROCK inhibitor, most of the pairwise correlations that existed between the local structural feature set (31–93) and global structural features (1–19), spectral features (20–30), local structural features (31–93) and shape-based morphological features (94–101) are altered.

**Figure 6:**
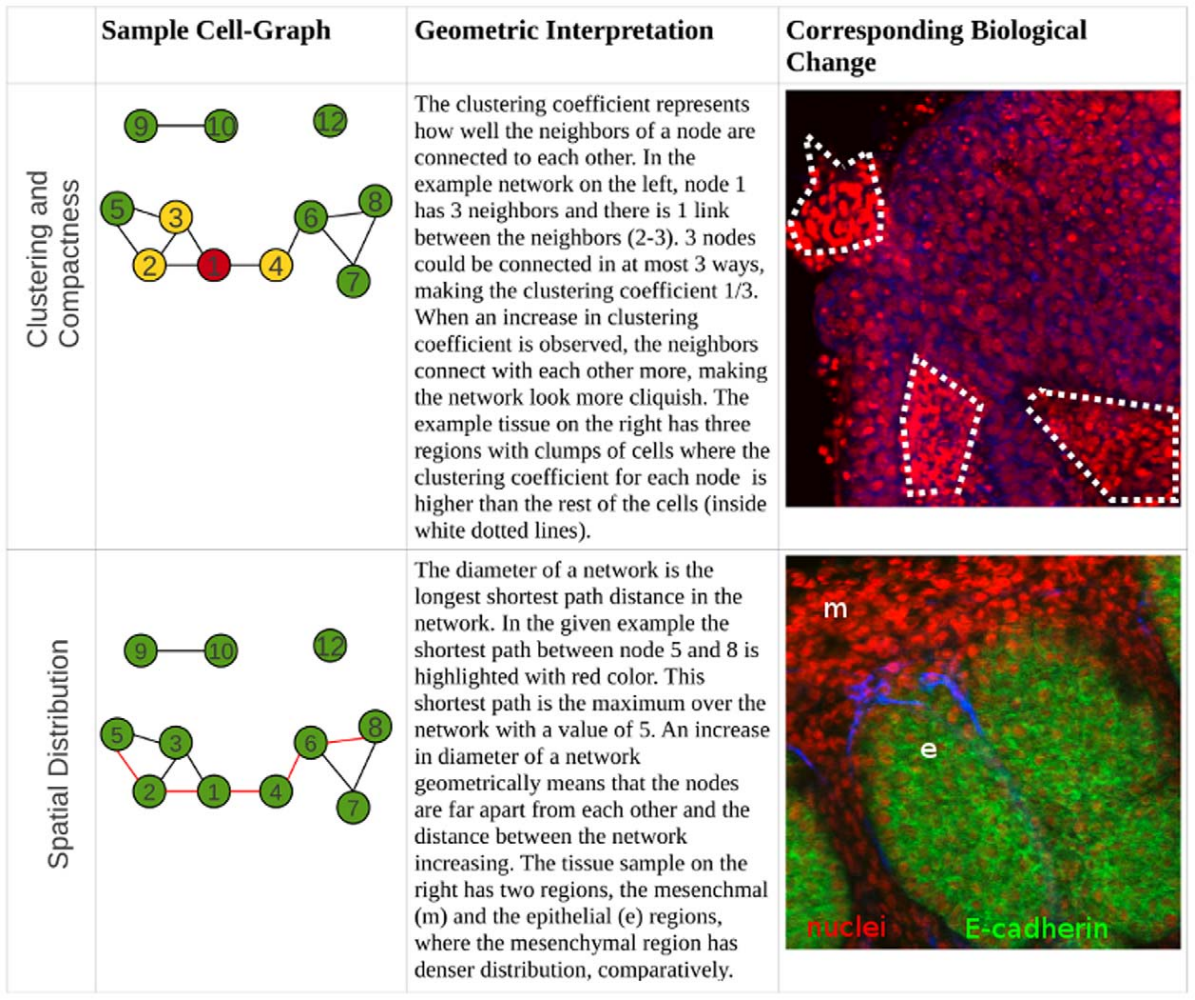
Geometric interpretation of changes in cell-graph features. A geometrical understanding of example cell-graph features is provided together with corresponding representative tissue samples. Geometrical interpretations of the changes for the example features are studied.

Feature correlation analysis in mesenchymal tissue: There are some similarities between the effect of ROCK inhibitor-treatment on mesenchymal and epithelial tissues. As in the case of the effect of ROCK inhibitor-treatment on the epithelial tissues, the pairwise correlations within the local structural feature set (31–93) are decreased for the inhibitor-treated mesenchymal tissues. rically, this means that the local behavior and characteristics of the nodes, or cells, in the network are different from each other; that is, they are more random with the treatment (Figure 6). Here, the number of pairwise significant correlations within the local structural feature set (31–93) decreases mainly for the features with indices 45–65 (Figure S4D). Furthermore, correlations within the global structural feature set decrease both in number and in value. A decrease in the correlation of global-structural feature set implies that the overall design principles of the network have been altered and the tissue samples do not resemble each other globally.

In the presence of the ROCK inhibitor in mesenchymal tissue, shortest path-related features (5–11) of the global structural feature set (1–19) become more correlated with the spectral feature set (20–30) and with the subset (40–50) of the local structural feature set (31–93) (Figure S4D). It is also important to note that significant correlations within the spectral feature set (20–30) become more uniform after ROCK inhibitor treatment (Figure S4D). As the shape-based features are calculated only for the epithelial tissues, no correlation changes for the shape-based morphological features are reported for mesenchymal tissues. Analysis of changes of correlations within and between the four feature categories suggests that inhibition of ROCK function affects epithelial and mesenchymal tissues through distinct mechanisms.

### Multi-way Tensor Analysis

**Figure 7:**
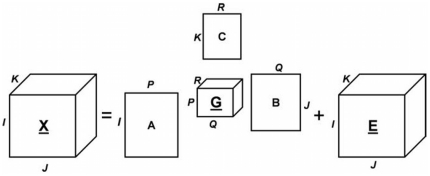
Illustration of a Tucker3 model for tensor analysis. 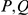
 **and** 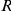 **indicate the number of components extracted from the first, second and third mode (** 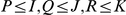 **), respectively, and** 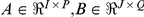 **and** 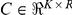 **are the component matrices.** 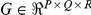 is the core tensor and 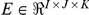 represents the error term.

We next performed multiway modeling and analysis [23] of our dataset that enables exploration of the data from different modes to model the dataset as a higher order array, to capture the multilinear structures in it, and to find underlying hidden patterns. Multiway arrays, often referred to as tensors, are higher-order generalizations of vectors and matrices. This modeling and analysis enables us to explore the features as well as the tissue types and the samples together by including each of these in the multiway analysis. In this analysis, we extracted 101 features from 20 tissue samples consisting of the four different tissue types: control epithelium, ROCK inhibitor-treated epithelium, control mesenchyme, and ROCK inhibitor-treated epithelium. This dataset is organized into a third order tensor of 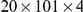 dimensions. A 3-way tensor can be modeled as shown in Figure 7.

**Figure 8:**
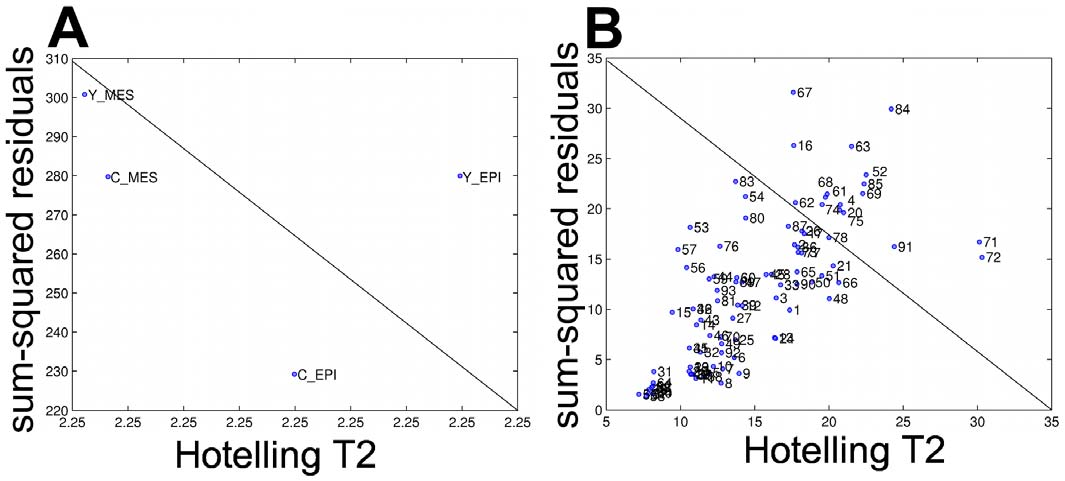
Multiway modeling by tensor analysis. Our dataset is modeled as a higher order array to capture the multilinear structures. (a) Tissue type analysis reveals that the untreated epithelial, untreated mesenchymal and treated mesenchymal tissues are grouped together. (b) Hotelling's T2 versus sum squared residuals to reveals features that the tensor analysis cannot fit with the model.

In the tissue type mode analysis, a clear differentiation between the treated and untreated samples exists, as shown in Figure 8A. From the sum-squared residuals vs the Hotelling's T^2^ value in Figure 8A, we can identify the tissue types that are distinct from the rest as those that appear above the diagonal line. Control epithelial and mesenchymal tissues and treated mesenchymal tissues, shown as “c_epi”, “c_mes” and “y_mes” respectively, are grouped all below this line whereas ROCK inhibitor-treated epithelial tissue, shown as “y_epi”, is scattered apart. We can conclude from this analysis that the ROCK inhibitor-treatment has a significant effect on the morphology and structure of the epithelial tissues and that the effect of the treatment is different on the epithelium and the mesenchyme, in support of the hypothesis that different cellular mechanisms are involved in development of each tissue.

Tensor analysis also provides information regarding the significance of each feature in the overall analysis. For instance, in features mode analysis, as displayed in Figure 8B in 2 dimensions from the sum-squared residuals vs the Hotelling's T^2^ value, we can identify all features that are distinct from the rest as those that appear above the diagonal line. One striking observation is that the skewness of the *knn* (k-nearest neighborhood) distances of all the representative nodes are chosen (61,62,63) as well as the mean of the physical *knn* distances of all the representative nodes (67,68,69). We also see that the standard deviation of the physical *knn* distance and the skewness of the physical *knn* distance of the second and the third representative nodes are identified by the tensor analysis. That is, k-nearest neighborhood-related features, which are in the local structural feature set, are identified by the tensor analysis.

### Supervised Learning of Control and ROCK Inhibitor Treated Tissues and Their Learning Accuracies

Next, we aimed to identify the minimal number of cell-graph features that could describe the structural difference between ROCK-inhibitor treated and untreated salivary glands. To accomplish this, we identified four tissue classification problems, control tissue classification (epithelium vs mesenchyme), ROCK inhibitor-treated tissue classification (epithelium vs mesenchyme), epithelial tissue classification (control vs ROCK inhibitor- treated), and mesenchymal tissue classification (control vs ROCK inhibitor-treated), and performed feature selection. Formally, feature selection is defined as the problem of reducing the dimensionality of the data to remove the irrelevant features and increase the learning accuracy. Since there are multiple feature selection algorithms available, and it is not readily apparent which would be most appropriate for our classification problems, we tested six different feature selection algorithms: no feature selection at all, relief method [24], symmetrical uncertainty attribute evaluation [25], consistency subset evaluation method [26], F-score feature selection [27], and correlation-based feature subset selection [28]. Since each of the selection algorithms uses different criteria for optimization, the features they select vary significantly. That is, a feature selection algorithm that tries to pick uncorrelated features e.g. consistency subset, might pick a different feature set than that of a feature selection algorithm that uses Fisher's criteria. Since the ultimate goal of any feature selection algorithm is to achieve the best learning accuracy using as few features as possible to define the problem, we reasoned that the features that were most often selected by the feature selection algorithms would be the optimal ones for recognizing the differences between the tissue states.

The four classification problems we defined were solved using support vector machines (SVM) and *K*-fold cross validation techniques, which have been used successfully for classification purposes [29]. SVM algorithm classifies the data by mapping it into a higher dimension and constructing an optimal separating hyperplane between data points such that the data points of different classes fall onto the opposite sides of this hyperplane. In the case that no such hyperplane exists (i.e. if the data is not linearly separable in this higher dimension), it constructs a hyperplane that leads to the least error. The learning accuracy of each solution is computed using the leave one out technique, which is a special case of *K*-fold cross validation technique. *K*-fold cross validation partitions the dataset into *K* disjoint subsets called folds. Of these *K* folds, *K-1* are used to train the model, and the remaining fold is used to test the model. This constitutes one iteration of the *K*-fold cross validation. Repeating this process *K* times, each time leaving out one fold for validation and using the other folds as the training set, the accuracy of each run is calculated and then averaged and reported as the cross validation accuracy. Typical choices for *K* are *K = 1*, *K = 5* and *K = 10*. When the data is limited in size, using leave one technique, *K = 1*, to ensure that enough data is used for learning is a common practice.

We examined the learning accuracy for the first two classification problems. For the first problem of distinguishing between control tissues (epithelium vs mesenchyme), the resulting learning accuracies for each of the six different feature selection algorithms are given in Table 5. All feature selection techniques, except the consistency subset evaluation technique, gave 100% learning accuracy. A similar test was performed for the ROCK inhibitor treated tissues (epithelium vs mesenchyme) and the learning accuracies for this second classification problem are given in Table 6. In this case, the best learning accuracy was also 100%, and 4 of the 6 feature selection algorithms achieved this accuracy. Thus, the multi-scale features, regardless of the feature selection method, are effective in distinguishing between the epithelial and mesenchymal tissue types, regardless of the inhibitor treatment. The obvious structural and morphological differences between the ROCK inhibited and untreated explants are reflected in the fact that this classification problem has 100% accuracy for almost all of the feature selection algorithms tried (Table 5 and Table 6).

**Table 5:** Epithelial vs Mesenchymal comparison in control tissue samples.

Feature Selection Algorithm	Selected Features	Best CV rate
SVM with No Feature Selection		100.0
SVM with F-score Selection	52,71,72,80	100.0
Correlation Based Selection	1,3,7,12,13,14,15,24,28,39,43,57,63,68,72,77,78,93	100.0
Relief Attribute Evaluation	39,52,71,72,80	100.0
Symmetrical Uncertainty	12,13,14,15,24,39,72	100.0
Consistency Subset Evaluation	12	97.5

**Table 6:** Epithelial vs Mesenchymal comparison in ROCK-inhibitor-treated tissues.

Feature Selection Algorithm	Selected Features	Best CV rate
SVM with No Feature Selection		95
SVM with F-score Selection	3,6,7,9,10,39,52,57,59,60,72,80,81,89,90	100.0
Correlation Based Selection	3,6,10,12,14,15,37,39,59,69	100.0
Relief Attribute Evaluation	7,39,56,57,59,60,80,81,89,90	97.5
Symmetrical Uncertainty	15,39	100.0
Consistency Subset Evaluation	15	100.0

The remaining sets of classification problems were designed to examine the effects of ROCK signaling within both tissue types. We separately compared the epithelial cell-graphs of the ROCK inhibitor-treated and control samples, as well as the cell-graphs corresponding to the mesenchymal tissues of inhibitor-treated and control samples. The results of the ROCK inhibitor versus control comparison for epithelial tissues are reported in **Table 7**. The best learning accuracy achieved was 100% achieved by the correlation-based subset evaluation technique and the symmetrical uncertainty technique. The mesenchymal comparison had 87.5% accuracy using the consistency subset and relief attribute evaluation technique **Table 8**. Not surprisingly, the cell-graphs were most effective in distinguishing between the two different tissue types. However, the cell-graphs were also able to distinguish between the control and ROCK inhibitor treated samples effectively, 100% and 87.5%, respectively.

**Table 7:** Control vs ROCK-inhibitor-treated comparison of epithelial tissues.

Feature Selection Algorithm	Selected Features	Best CV rate
SVM with No Feature Selection		100.0
SVM with F-score Selection	1,3,15,21,68,99	95.0
Correlation Based Selection	1,3,15,65,68,92,99	100.0
Relief Attribute Evaluation	1,3,15,21,32,41,50,56,92,98,99,100	97.5
Symmetrical Uncertainty	1,3,15,65,68,92,99	100.0
Consistency Subset Evaluation	1,3,65	92.5

**Table 8:** Control vs ROCK-inhibitor-treated comparison of mesenchymal tissues.

Feature Selection Algorithm	Selected Features	Best CV rate
SVM with No Feature Selection		72.5
SVM with F-score Selection	1,2,3,15,20,21	80.0
Correlation Based Selection	1,2,3,21,55,65	80.0
Relief Attribute Evaluation	1,2,3,4,15,20,21,83,91	87.5
Symmetrical Uncertainty	1,2,3,21,65	82.5
Consistency Subset Evaluation	2,3,65	87.5

### Feature Selection

The features selected by the feature analysis algorithms provide informative quantitative descriptions of alterations in cellular and tissue-level changes for each classification problem. For the six feature selection algorithms explored in the epithelial vs mesenchymal analysis in the untreated samples, features indexed with 12, 39 and 72, which are giant connected component, clustering coefficient of the third representative node and the standard deviation of the *knn* distance of the same representative node, were commonly selected by the algorithms as the minimum number of features needed to distinguish between these two tissue types, see Table 5. In this classification, a global and two local structural features were found to be the most informative.

In the presence of the ROCK inhibitor, features indexed with 15, 39 and 59, which are namely: percent of end points, local clustering coefficient and standard deviation of the *knn* distance are the most informative features, as shown in Table 6. The local clustering coefficient measures cliquishness and the connectivity of the tissues and gives a measurement of how addition of ROCK inhibitor affects the connectivity of the epithelium and mesenchyme differentially, which was not previously known. Also in the ROCK inhibitor-treated case, the percent of end points, which are the nodes that have only one neighbor, and that also measures the connectivity of the tissue was important. These findings are consistent with the biological observation that the cellular organization of epithelium is different than the cellular organization of the mesenchyme.

The comparison in which we expected to observe the greatest change in tissue shape based on biological studies [19], [20], [21], was the comparison between control- and ROCK inhibitor-treated epithelium. We previously demonstrated that SMGs treated with ROCK inhibitors showed an inhibition of branching morphogenesis, which was associated with a distended appearance of the gland [19], measureable as an increase in the tissue diameter (**Figure 4**A). When ROCK inhibitor-treated epithelial tissue was compared to control epithelial tissue, the features 1,3,15,65,68,92 and 99 were identified, where 1,3,15 are average degree (1), clustering coefficient (3) and percent of end points (15) (Table 7). Interestingly, all of the feature selection algorithms selected average degree and global clustering (1,3). The difference in cellular compactness predicted by the cell-graphs is significant and consistent with the biological predictions. Alteration of cellular connectivity is thus a direct effect of the ROCK inhibitor in epithelial tissue, as expected based on previous results ([21]). The rest of the selected features were local features representing the *knn* distances between the cells and the number of hybrid edges. This suggests that some form of epithelial-mesenchymal interactions captured by the hybrid edges, are regulated by ROCK1. Also in the control- versus ROCK inhibitor-treated epithelial tissue comparison, three out of the five feature selection algorithms selected the 99th feature. The feature indexed with 99 is a shape-based feature, thus taking into account the change in overall shape of the explants upon addition of the ROCK inhibitor. This is consistent with the comparison of the perimeter values calculated by the cell graphs (Figure 4E) and by conventional image analysis (Figure 4B).

When mesenchymal tissue was compared between ROCK inhibitor-treated and control explants, the feature selection algorithms that achieve the highest accuracy picked features 2 and 3 in common, which are the two different global clustering coefficients. Cell-graphs thus predict that previously unappreciated differences in cellular compactness are also significant in ROCK inhibitor treated versus control treated mesenchymal tissue. Interestingly, four of the feature selection algorithms picked a spectral feature (21) and three of them picked the kurtosis of the *knn* distance. Significantly, features derived from different scales were important in the mesenchymal tissue comparison.

### Multiscale Feature Analysis

Since the feature selection algorithms selected features representing multiple categories (Table 1, 2, 3,4) for the defined classification problems, this result implied that multiscale feature analysis is advantageous for identification and classification of tissues, moreso than unidimensional cell-graph analysis. To confirm this hypothesis, for the four classification problems defined previously, we calculated the learning accuracy using only the global graph features, local graph features, spectral features, or morphological features, and compared these results with those obtained using all classes of features, which we defined as multiscale feature analysis. Using multiscale feature analysis, the learning accuracy was the highest for all of the classification problems (Table 9).

**Table 9:** Comparison of the learning accuracies using all the multi-scale features or only global graph features, spectral features, local graph features or morphological features.

Feature Selection Algorithm	Multiscale Feature Set	Global Graph Features	Spectral Features	Local Graph Features	Shape Based Features
Epithelial vs Mesenchymal in control	100	100	97.50	100	-
Epithelial vs mesenchymal in ROCK treated	100	97.50	90	95	-
ROCK Treated vs control in epithelial	100	97.50	77.50	82.50	90
ROCK treated vs control in mesenchymal	87.50	85.00	77.50	70	-

For the epithelial versus mesenchymal comparison in control samples, the accuracy was 100% using the multiscale set of features, which was also achieved by local structural features and the global features. In ROCK-inhibitor-treated epithelium versus mesenchyme, multiscale feature analysis also achieved 100% accuracy, followed by 97.5% accuracy of global structural features alone. In ROCK inhibitor-treated vs control for epithelial tissue samples, multiscale feature analysis was able to achieve again 100% learning accuracy, which was not achieved by any of the other set of features alone. The closest accuracy was performed again by the global structural graph features alone with 97.5% accuracy. In this comparison, shape-based morphological features alone achieved 90% accuracy. In ROCK inhibitor-treated versus control mesenchymal tissues, multiscale features were 87.5% effective, but none of the individual feature sets were able to achieve a better accuracy. From these analyses, we conclude that multi-scale feature analysis achieves the highest levels of accuracy for discriminating between tissue types and is more effective than any type of unidimensional cell-graph analysis group.

## Discussion

We report utilization of a novel multiscale feature analysis to capture morphological and cellular changes accompanied with perturbation of a ROCK1-mediated signaling pathway in both epithelial and mesenchymal tissue types in developing salivary glands. Using six different feature selection algorithms, we identified specific subsets of features that most efficiently and effectively define differences between ROCK inhibitor-treated vs control glands in two tissues at multiple biological levels and at local and global scales. Tensor analysis demonstrated that the ROCK inhibitor affects the epithelial tissues the most significantly. Clustering analysis revealed significant correlations between structural, morphological, local and spectral features. Similarly, comparison of multiscale feature analysis vs unidimensional analysis revealed that a multiscale feature analysis more accurately models each tissue under both conditions than does any uniscale analysis.

Through this study we identified a multiscale features signature for both epithelial and mesenchymal salivary gland tissues and a specific ROCK inhibitor-induced signature for each tissue type. Some of the cell graph features provide insights into specific biological parameters. That cell graphs can distinguish between the different cell types (epithelium and mesenchyme) is interesting and significant, especially since these cell populations are complex. The embryonic epithelial cell type described here is assumed to be composed of equivalent cells at this early stage of development and will later develop into saliva-secreting acinar cells, saliva-producing and modifying ductal cells, and tissue regenerating progenitor cell populations. The mesenchymal cell compartment at these developmental stages is complex and includes fibroblasts, neuronal cells, and arterial cells. It will be interesting to use cell-graphs of increasing complexity to distinguish between these distinct cell sub-populations in future studies. That cell-graphs can distinguish between cell populations that have been treated with ROCK inhibitors is significant. The features that were selected using feature analysis indicate that the epithelial cell clustering, or cell spacing, increases in the absence of ROCK signaling. Biologically, this could be indicative of a decrease in cell-cell adhesions, an increase in cell size, or decrease in proliferation. In light of previous research indicating that ROCK affects cellular contractility and cell proliferation [19], the change in cell shape is most likely. Interestingly, recent work indicates that effects of ROCK inhibitor are context dependent and that basement membrane prevents ROCK signaling from affecting cell-cell adhesions in epithelial cells that contact it. It will be interesting to use cell-graphs to examine sub-sets of cell populations at distinct time periods in future studies.

Since this study has demonstrated the utility of the cell-graph modeling for understanding organogenesis at multiple biological scales, future studies can address the specific contributions of additional signaling pathways, which would be predicted to have their own signature for each specific tissue type. Branching morphogenesis is a dynamic process leading to establishment of a functional tissue, and it is likely that each developmental stage will have a specific cell-graph signature. In future studies that will model the process of branching morphogenesis over time, the challenge will be to integrate cellular dynamics, tissue-level patterning events, with the intercellular signaling mechanisms across multiple time scales in dynamically changing cell populations. By developing a cell-graph signature for each incremental change in tissue state when specific signaling pathways have been disrupted, cell-graphs will provide a quantitative method to facilitate building of multiscale cell-based simulations of organ development.

The cell-graph analysis presented here represents the first attempt to model cellular behavior as a component of the process of branching morphogenesis. Lung morphogenesis was modeled previously using a continuous free-boundary method in which only two factors influencing the boundary were considered: diffusion of growth factors and concentration of nutrients [30]. Previous studies modeling salivary gland morphogenesis focused on hypothetical modeling of physical forces, based on physical parameters, such as tissue viscosity, and neglected a cellular component. In one study, a two-dimensional model of salivary gland branching morphogenesis was generated where epithelium and mesenchyme were modeled as immiscible Stokes fluids of constant viscosities [31]. Later, a three-dimensional model was developed in which mesenchymal cells were more realistically considered using fluid mechanics to model hypothesized mesenchyme-generated traction forces [32]. Notably, in these studies, cells were not accurately modeled and the actual shape of the epithelial tissue was not achieved by the mathematical simulations, leaving open the possibility that accurate modeling of cellular rearrangements may contribute significantly to a realistic model. The most realistic models of branching morphogenesis will incorporate physical, cellular, and molecular signaling to create both descriptive and predictive models.

Mathematical modeling is currently being employed to better understand and predict multiple biological problems [33]. This study provides the first step towards using “multiscale feature analysis” to understand development of complex branching tissues. If we can understand the design principles that govern biological organization locally and globally and understand the correlation between molecular signaling, cellular response, and structural and morphological alterations in the tissue, these principles can be used to inform and accelerate studies of tissue morphogenesis, development, differentiation, and organ formation. Thus, we present the utility of multiscale feature analysis in developing salivary gland tissue, suggesting that these methods will be useful in future modeling efforts of this complex process of branching morphogenesis and other developmental processes.

## Materials and Methods

### Organ Culture and Inhibitor Treatment

Ex vivo organ culture: Mouse SMGs were dissected from timed pregnant female mice (strain CD-1, Charles River Laboratories) at embryonic day 13 (4 to 5 buds), with the day of plug discovery designated as E0, following protocols approved by the University at Albany IACUC committee (protocol 09–013). SMGs were microdissected from mandible slices and cultured, as described previously [19], [34]–[37]. SMG organ cultures were exposed to the ROCK inhibitor dissolved in culture media at 140 µM (Y27632, 6888000 Calbiochem), or vehicle control media for 24 hrs prior to fixation in 4% paraformaldehyde in 1× phosphate buffered saline (PBS), as described previously [19]. Greater than 20 SMG organ explants were included in each treatment group.

### Whole-Mount Immunocytochemistry

Whole-mount immunocytochemistry was performed using SMGs fixed in 4% paraformaldehye (PFA) in 1× phosphate-buffered saline (1XPBS) containing 5% (w/v) sucrose for 20 min at room temperature. SybR Green I (Invitrogen) was used to detect nuclei. Epithelium was detected using an antibody recognizing E-cadherin (1∶100, BD Biosciences) and a Cy3-conjugated Donkey F(ab)_2_ secondary antibody (1∶100, Jackson ImmunoResearch Lab).

### Confocal Imaging

Immunostained glands were imaged using a laser scanning confocal microscope (Zeiss 510 Meta) at 20× (Plan APO/0.75 NA) using identical settings for all samples. Multiple images overlapping each other by approximately 10% were acquired at the center of each explant (Z-dimension) such that the entire explant was imaged.

### Image Registration

To image complete biological specimens with relatively high resolution, images having overlapping regions were captured and computationally stitched together. For stitching, the same approach described as in [38] was followed. A correlation matrix 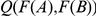 of the phases of the Fourier transforms of the images was calculated. The peaks of the inverse Fourier transform of the phase correlation matrix, 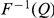, give highly correlated regions of the images. In cases in which there was more than one highly correlated region in the image, the correct shift was found by cross-correlation on the overlapping areas of the input images. After finding the correct shift, a non-linear blending image fusion technique was also performed to compensate for intensity non-uniformity in the source images.

### Image Segmentation

Prior to image segmentation, stitched images were examined manually, to exclude whole or partial sublingual glands that were included in the image by manually drawing a line in the mesenchyme to separate the two organs Figure S2. The submandibular gland image was kept and the 20 most representative images containing non-damaged submandibular glands were included in the subsequent analysis.

A coarse initial segmentation of the epithelium was performed using the Otsu thresholding algorithm [39]. The result of this step was then used for the initialization of the active contours without edges technique [40]. In the active contours approach, the image f_0_ is assumed to be formed by two regions of approximately piecewise-constant intensities f_0_^o^, f_0_^i^ corresponding to inside and outside of a curve C in Ω_,_ where ω denotes the region inside the curve C, and 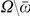 denotes the region outside the curve C. Using the area and the length of this curve as regularization terms, the Chan and Vese approach introduces the following energy functional
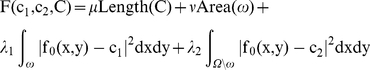
and solves the problem of minimizing it over the curves C and the average intensity values 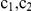 inside and outside the curves, where 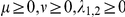 are fixed parameters used for weighting the length, the area, the average intensity inside the curve, and the average intensity outside the curve respectively. We set these parameters equal to 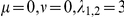.

The Otsu algorithm, followed by morphological watershed technique was used to segment the nuclei [22],[39]. The Otsu thresholding algorithm assumes that there are two classes of pixels in the observed image and finds a threshold value that will automatically separate the foreground pixels (defined as epithelium by the presence of E-cadherin signal) from the background (defined as lacking E-cadherin signal). The algorithm searches for the threshold value that minimizes the intra-class variance. After finding this optimal threshold, the intensity values of each pixel were compared against the threshold and the pixels with intensity values higher than the threshold were assigned as foreground pixels. Using the mask obtained, the nuclei were marked as epithelial nuclei or mesenchymal nuclei depending on whether they resided in the mask or not.

### Conventional Image Analysis

For biological validation, MetaMorph Advanced (Version 7.7.0.0) (Molecular Devices Inc.) was used to measure diameter, area, and perimeter of the explants using calibrated confocal images imported into the program as tiff files. An outline of the gland was manually drawn and thresholding was performed to include the highest number of pixels in that region. The region statistics tool was used to calculate area, perimeter, and the diameter of the images. Circularity was calculated based on the formula:
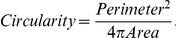



### Cell-Graphs

The colored cell-graph construction algorithm [13],[16] was used to model the structure of the salivary gland tissues. In colored cell-graphs, a relationship between two vertices is hypothesized when those vertices are touching or if they are close to each other and they are of same tissue type. Biologically, an edge between two vertices might mean that these cells are touching to each other and are connected by cell-cell adhesions. In salivary gland modeling, two different cell-graphs, one representing epithelial and the other representing mesenchymal tissues, are built capturing the spatial organization of cells in the two tissue comparments. We find the center of mass for each nucleus and store the x-y coordinates. We hypothesize a communication by setting a link between two vertices if the Euclidean distance between them, 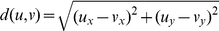, is less than a threshold that ensures a physical contact between the corresponding cell membranes.

The choice of distance threshold was determined by using a cross-validation technique whenever possible. In K-fold cross validation, the dataset is divided into K folds, and of these K folds, K-1 are used to train the system using the specific threshold. The accuracy is then calculated by testing the learning system on the remaining fold. Free parameters such as distance threshold can be accurately found and set using K-fold cross validation. The cell-graph representation of the tissue was also visually compared to the original confocal images to confirm that the resulting cell graph was reflective of the images in our analysis.

### Feature Extraction

In our previous work, we primarily focused on the global structural modeling of the tissues and therefore did not include shape-based or local-structural features [11]–[16]. Here, in this study, to capture changes that take place at different scales of the salivary gland we also calculated shape metrics and extracted features representing local properties. As a result, our feature set consists of four different types of features namely global-structural, local-structural, morphological/shape-based, and spectral features.

#### Global Structural Features

A variety of features were calculated from the spatial distribution of the cells (Table 1). The simplest features are those defined by counting the number of cells and the number of communication links between the cells. The average number of communication links gives the average degree metric. Giant connected component, number of connected components, and percentage of isolated points features quantify the connectedness and denseness of the tissue. Features that quantify how far the vertices are apart from each other are also calculated. The shortest path between two vertices is defined as the minimum number of hops between them. Using this definition, the eccentricity of a node 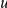 is given as the maximum shortest path distance from node *u* to any of the vertices in the graph. After the calculation of the eccentricities of each node, the diameter of the graph is simply given by the maximum eccentricity. The minimum eccentricity is defined as the radius feature, and the vertices that have eccentricity equal to the radius are called central points. The complete list of these features is given in Table 1 and a geometric understanding of the features are provided in Figure 6.

#### Spectral Features

The spectral analysis of graphs [41] focuses on the eigenvalues of the matrix representation of the graphs and gives insight into structural organization of the tissue. A biological explanation of each and every spectral metrics is not always possible though some of them have direct explanations. For instance, the number of zero valued eigenvalues in normalized Laplacian matrix gives the number of connected components. We included spectral features extracted from both the adjacency and normalized Laplacian matrices. The normalized version of the Laplacian matrix reads
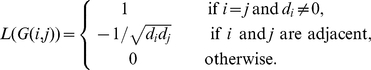
The spectral decomposition of the normalized Laplacian matrix,
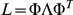
_,_ where 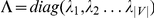 is a diagonal matrix having the eigenvalues of 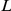
as its elements and 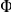 is a matrix having the eigenvectors of 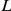
as its columns. Since 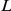 is a symmetric, positive semi-definite matrix, all the eigenvalues of the normalized Laplacian matrix 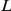 lie between the values of 0 and 2.

For normalized Laplacian matrices, the number of zero eigenvalues gives the number of connected components in the graph. We included the number of zero eigenvalues, the number of eigenvalues equal to one, and the number of eigenvalues equal to two in our feature set. We sort and plot the eigenvalues of the normalized Laplacian matrix in an increasing order and then fit a line to this plot in a least squares manner. The slope of the line for the eigenvalues that are between 0 and 1 is called the lower slope. Likewise, the upper slope is defined as the slope of the eigenvalues between 1 and 2.

The last two normalized Laplacian matrix features we include in our feature set are the trace of the normalized Laplacian and energy of the normalized Laplacian, defined as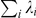 and 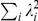. A summary of these features and their explanations are given in Table 2.

**Local Features:** We extracted specific features to analyze the local behavior of the collected tissues (Table 3). We extracted 21 features for every node in the cell-graphs. Some simple features include the degree of a node, given by the number of nodes that it is connected to. We calculated features capturing pairwise hop distances, such as the eccentricity value, given as the maximum value of the shortest path values from the node. Clustering and connectedness features of a node such as the clustering coefficient, calculated as the ratio of the links of the node's neighbors have in common to the total number that can possibly exist, are also included in this analysis. We also included features capturing the physical distances of the neighborhood of a node. The mean of the physical distances of a node's neighbor is called the mean edge length and the mean of the physical distances between a node and the nodes that are at k times the link threshold distance from that is assigned as the mean physical distance of k-nearest neighborhood.

In our local representation of the tissue, we have an Nx21 matrix where the rows are the vertices of the graphs and the columns are the features for each node. There are three types of node clusters in epithelial tissues, namely border cells, bud cells, and duct cells. Therefore, we could cluster and represent our local features matrix with a 3×21 matrix, giving a total of 63 elements. That is, each of our tissues can be represented by 63 features to perform local analysis. We call the rows of this reduced matrix, cluster 1, cluster 2, and cluster 3. The rows can also be thought of as the representative vertices. As before, the columns of this matrix refer to the features.

#### Morphological Features

Shape-based features, given in Table 4, of epithelial tissues and mesenchymal tissues were calculated. The simplest of these shape-based metrics are the area, *A*, and the perimeter, *P*, of the tissue. The major and the minor axis upon which the tissue lies were calculated, and the ratio of the length in each direction was calculated to give the tissue elongation. For circular tissues, the elongation should be close to 1 whereas for a tissue that looks like an ellipse, the elongation is greater than 1. A commonly used shape-based feature is the solidity of the region. To calculate the solidity of a region, the convex hull of the region is found and the ratio of the tissue area to the convex hull area is calculated. This feature gives how convex the region is, and therefore is also referred to as the convexity. Another commonly used feature is the circularity of the region. Circularity is simply calculated by 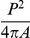. A full list of the shape-based features is provided in Table 4.

### Feature Correlation

A simple measure to calculate the dependencies between features 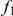 and 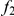
is the Pearson correlation coefficient. The Pearson correlation coefficient measures how features correlate with respect to each other and takes values between −1, representing a perfect negative linear dependency and +1, representing a perfect positive linear dependency. When two features vary independently of each other, Pearson correlation coefficient takes the value 0. The Pearson correlation coefficient is computed according to the formula 
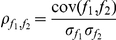
 where 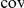 is the covariance of the features 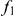
and 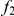, and 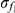 and 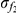 are the standard deviations of 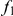 and 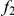.

### Multiway Modeling

We extracted 101 features for 4 different tissue types, namely control epithelial, control mesenchymal, inhibitor-treated epithelial and inhibitor-treated mesenchymal. This dataset is organized into a third order tensor of 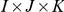 dimensions. An entry 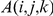 in the cube is the value of feature j, for sample i, of the tissue type k where 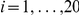; 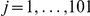; and 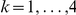. We used one of the most common multiway analysis techniques (the Tucker3 model) [23]. A 3-way tensor 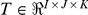, where 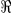 denotes the set of real numbers, using a Tucker3 model was modeled as in equation 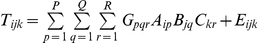
where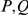 and 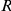 indicate the number of components extracted from the first, second and third mode (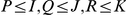), respectively, and 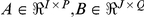 and 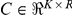 are the component matrices. 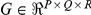 is the core tensor and 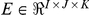 represents the error term.

Model fitting was calculated for normalized (zero mean, unit deviation) data with the core tensor of dimensions 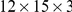 yielding an accuracy of fitting as 85.167% using Tucker3 decomposition. The analysis focused on the feature mode to identify a subset of the cell-graph metrics that are more influential than the others to explain the 3-way data. We have used the Hotelling's T^2^ statistics built in the MATLAB PLS Toolbox 4.0 and MATLAB Tensor Toolbox implemented by Brett W. Bader and Tamara G. Kolda at SANDIA [42].

### Feature Selection

Feature selection can be defined as the problem of reducing the dimensionality of the data to remove the irrelevant data and to increase the learning accuracy. Several feature selection algorithms have been proposed, and a detailed survey is given in [43]. We used the Weka software [44] to test various feature selection algorithms.

#### Symmetrical Uncertainty Attribute Evaluation

In the Symmetrical Uncertainty Attribute Evaluation method [25], a feature is considered “good” if it correlates well with the class label but does not correlate with any other “good” features. There are two ways to measure correlation between two random variables: classical linear correlation (which we provide in Figure S3) and information theoretic entropy-based correlation. The proposed method, Symmetrical Uncertainty, uses information theory to design a correlation based feature selection algorithm. The authors used the concept of symmetrical uncertainty to devise their algorithm. Symmetrical uncertainty between two random variables is given by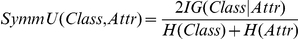. Here 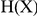 is the entropy of the random variable X, 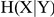 is the entropy of X given the random variable Y and 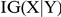 is the information gain that measures the change in the entropy of X given the random variable Y computed respectively as
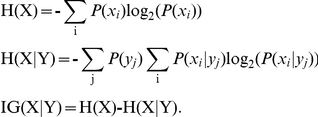
Using these definitions, the algorithm is given as follows. First, features with high SU values are found and ranked according to their SU values. Then, these features are further investigated in the order of their SU values, and the redundant features (features that are correlated with others that have higher SU values) are discarded.

#### Consistency Subset Evaluation Method

The authors introduced a probabilistic approach to feature selection in [26] by using a Las Vegas Algorithm. In every round a random subset, S, from M features is generated. If this new subset of features has cardinality less than the current best feature set and the inconsistency value of this subset is lower than a predefined threshold, this new set becomes the current best subset.

The success of the algorithm depends on the definition of the inconsistency criterion. This criterion has two parts to it. First, if two instances match except for their class labels, they are considered to be inconsistent. Second, the inconsistency count is defined as the number of matching instances minus the largest number of instances of class labels [26].

#### F-score Feature Selection

The authors introduce a feature selection method based on the Fisher's criterion [45] in [27]. This method assigns each feature the associated Fisher score and ranks them in descending order. F-score gives discriminative capability of each feature, i.e. when feature f is more discriminative, the associated F-score 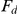 is larger. For a dataset X with two class labels, denote the instances in class 1 with 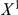 and instances in class 2 with 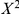. The Fisher score of the i^th^ feature is then given by
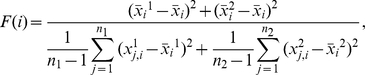
where 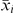 is the average of feature i in the whole data set and 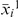, 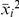 are the averages of the i^th^ feature, for instances with class 1 and class 2 labels respectively. 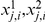 are the i^th^ feature of the j^th^ instance for class 1 and class 2, respectively, and n_1_ and n_2_ are the number of instances for each class.

#### Correlation-Based Feature Subset Selection

A correlation-based feature selection algorithm that ranks feature subsets according to a correlation-based heuristic is introduced in [28]. Using this heuristic, subsets containing features that are highly correlated with the class label and uncorrelated with each other are chosen. The goodness,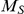, of a given subset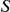 is measured as
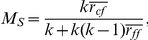
where k is the number of features, 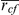 is the mean feature-class correlation and 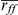 is the feature-feature inter-correlation.

#### Relief Attribute Evaluation Feature Selection

Relief attribute evaluation repeatedly samples an instance and considers the value of the given attribute for the nearest instance of the same and different class [24]. The algorithm sorts the features according to their weights calculated as shown in the following algorithm;

set all weights W[A]: = 0.0

for i: = 1 to m do

 randomly select an instance R;

 find nearest hit H and nearest miss M;

 for A: = 1 to all attributes do

  W[A]: = W[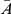]-diff(A,R,H)/m+diff(A,R,M)/m.

Here, diff (Attribute, Instance1, Instance2) measures the difference between the values of the specific attribute for two instances and H and R are the nearest instance of the same and different class.

## Supporting Information

Figure S1 **The outer layer of epithelial cells is disorganized in the presence of ROCK inhibitor.** Confocal images were captured of SMGs treated with (a) control media or (b) ROCK inhibitor and immunostained with E-cadherin (red) to label epithelium and Sybr Green (green) to label nuclei. The control SMGs show an outer layer of epithelial cells that is highly ordered (as marked with a dotted line below this cell layer) whereas the ROCK inhibitor-treated SMGs do not have this highly ordered cell arrangement (arrows). Scale,50 mM.
(TIF)

Figure S2 **Sublingual and submandibular glands are depicted. In our analysis, we manually discarded the sublingual regions of the samples, depicted with the dashed region in the figure, and only used the submandibilar glands.**
(TIF)

Figure S3 **Feature correlations for different tissue types are shown.** Cell-graph feature correlations were clustered into four groups using the k-means clustering algorithm. Features that are highly correlated are grouped together. In (a) control epithelial tissues, (b) ROCK-inhibitor-treated epithelium, (c) control mesenchymal tissue, and (d) ROCK inhibitor treated mesenchymal tissue correlation clusters are depicted.
(TIF)

Figure S4 **Statistically significant pair-wise correlations.** Absolute values of the significant correlations for control epithelial tissues are shown in (a), ROCK inhibitor-treated epithelial tissues are shown in (b), control mesenchymal tissue in (c) and ROCK inhibitor-treated mesenchymal tissue in (d). Features are shown in numerical order.
(TIF)
