# Supplementary figures and images for: Multiscale Feature Analysis of Salivary Gland Branching Morphogenesis
Source: PLoS One. 2012 Mar 5;7(3):e32906. doi: 10.1371/journal.pone.0032906 (PMC3293912; doi:10.1371/journal.pone.0032906)

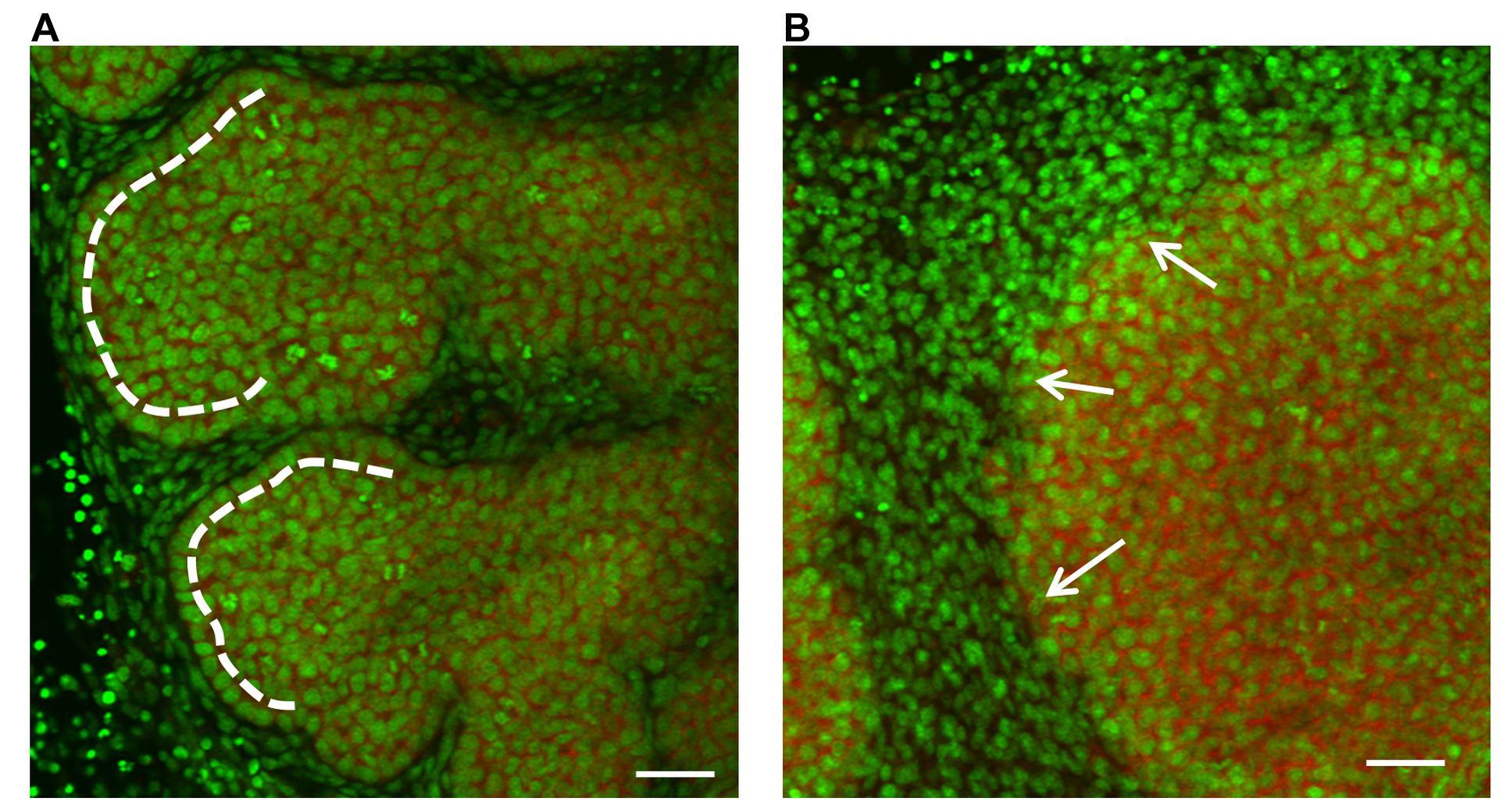

Supplement: Figure S1 — The outer layer of epithelial cells is disorganized in the presence of ROCK inhibitor. Confocal images were captured of SMGs treated with (a) control media or (b) ROCK inhibitor and immunostained with E-cadherin (red) to label epithelium and Sybr Green (green) to label nuclei. The control SMGs show an outer layer of epithelial cells that is highly ordered (as marked with a dotted line below this cell layer) whereas the ROCK inhibitor-treated SMGs do not have this highly ordered cell arrangement (arrows). Scale,50 mM. (TIF) [file pone.0032906.s001.tif]

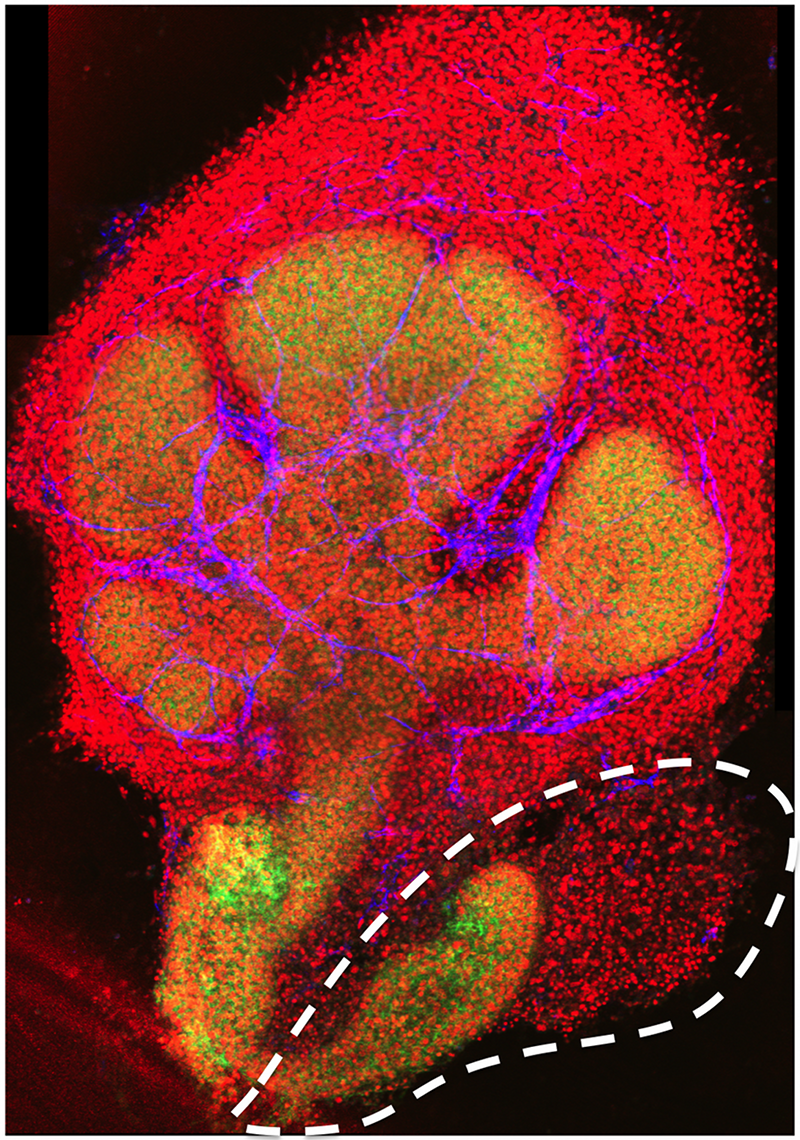

Supplement: Figure S2 — Sublingual and submandibular glands are depicted. In our analysis, we manually discarded the sublingual regions of the samples, depicted with the dashed region in the figure, and only used the submandibilar glands. (TIF) [file pone.0032906.s002.tif]

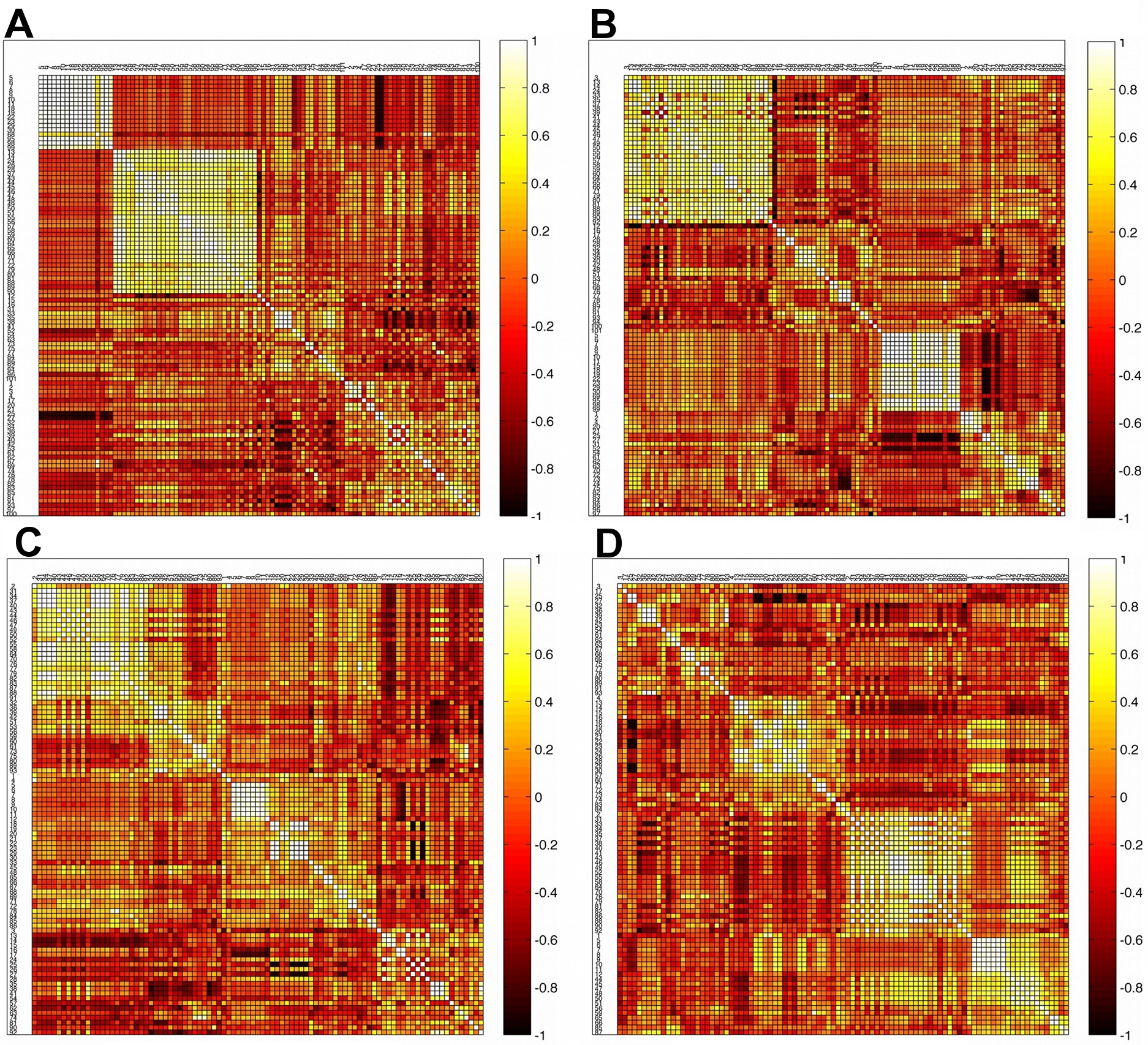

Supplement: Figure S3 — Feature correlations for different tissue types are shown. Cell-graph feature correlations were clustered into four groups using the k-means clustering algorithm. Features that are highly correlated are grouped together. In (a) control epithelial tissues, (b) ROCK-inhibitor-treated epithelium, (c) control mesenchymal tissue, and (d) ROCK inhibitor treated mesenchymal tissue correlation clusters are depicted. (TIF) [file pone.0032906.s003.tif]

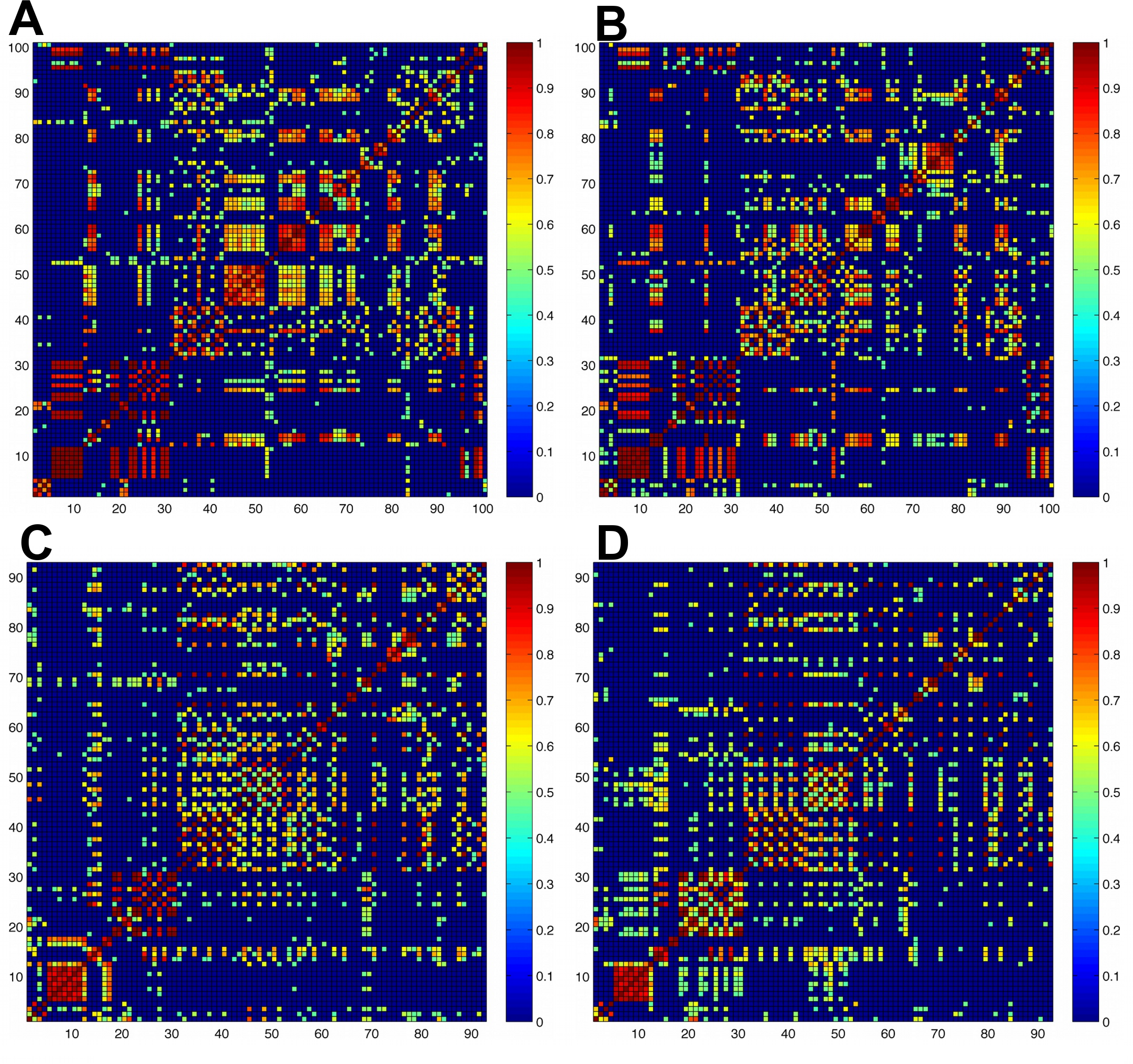

Supplement: Figure S4 — Statistically significant pair-wise correlations. Absolute values of the significant correlations for control epithelial tissues are shown in (a), ROCK inhibitor-treated epithelial tissues are shown in (b), control mesenchymal tissue in (c) and ROCK inhibitor-treated mesenchymal tissue in (d). Features are shown in numerical order. (TIF) [file pone.0032906.s004.tif]
